# Incidence and Outcomes of Emergency Presentation With Complicated Abdominal Wall Hernias: A Retrospective Study

**DOI:** 10.7759/cureus.75688

**Published:** 2024-12-14

**Authors:** Kehkashan Anwar, Sourabh Jadhav, Jahnavi Pasila, Muhammad Talal Nasir, Andrei Mihailescu

**Affiliations:** 1 General Surgery, Tameside General Hospital, Manchester, GBR; 2 Colorectal Surgery, Tameside General Hospital, Manchester, GBR

**Keywords:** femoral hernia, hernia, incarceration, inguinal hernia repair, laparoscopic vs open, post-op complications, strangulated inguinal hernia, strangulation, umbilical hernia, umbilical hernia repair

## Abstract

Aims

This study aims to analyse the type of repair and post-op outcomes for individuals who underwent surgical intervention and presented with symptomatic abdominal wall hernia as an emergency. It highlights the importance of timely elective hernia management in lowering emergency presentations and any related complications.

Background

Abdominal wall hernias are common surgical conditions that can present electively or as emergencies, with emergency cases being associated with higher complication rates. In recent years, there has been an increase in emergency hernia presentations, leading to a greater number of urgent surgeries. These emergency operations have been linked to higher rates of post-op complications and re-interventions due to factors like bowel obstruction, strangulation, and delayed intervention. Therefore, research and analysis of post-op outcomes are essential to highlight the potential role of elective hernia management in reducing the burden of emergency cases.

Methods

This study was a single-institution retrospective study that looked at patient data over a 10-year period and involved patients who presented with abdominal wall hernias on an emergency basis and underwent surgical repair. Data were collected on patient demographics, presenting symptoms, hernia type, surgical technique, use of mesh reinforcement, post-op complications, re-intervention rates, and length of hospital stay. The database was developed and analysed with IBM SPSS Statistics for Windows, Version 26 (Released 2019; IBM Corp., Armonk, NY, USA), and the results were considered significant at p<0.005.

Results

Over a 10-year period, 239 patients presented with emergency abdominal wall hernias, with 238 undergoing surgical repair; one patient died before surgery. Most repairs were performed using an open approach (93%, n=221), while 7% (n=17) were laparoscopic. Primary repair was conducted in 47% of cases (n=111), and mesh reinforcement was used in 53% (n=127). The overall recurrence rate was low (2%, n=4), with higher recurrence observed only in primary repairs. Mesh repairs had a higher incidence of post-op complications (56%, n=37) compared to primary repairs (44%, n=29). Patients who underwent mesh repair had an average hospital stay of eight days compared to those with primary repair (nine days). Inguinal, umbilical, and femoral hernias were the most common types observed, accounting for over 76% of cases. In total, 6% of patients (n=15) required bowel resections, highlighting the complexity of these cases. Most patients (72%, n=172) experienced no complications, but some of the most common post-op complications were wound dehiscence (7%, n=17), post-op collection (7%, n=16), delayed recovery (3%, n=8), and recurrence (2%, n=4).

Conclusion

It has been observed that a greater number of post-op complications and longer hospital stays are linked to emergency hernia procedures, especially when mesh repair is utilised. Although the recurrence rate of mesh repairs is lower than that of primary repair, the increased risk of complications highlights the significance of cautious surgical planning and patient selection. In conclusion, this study highlights the benefits of elective hernia management in reducing emergency presentations and the unfavourable consequences that may arise from it. These results support the importance of pre-op optimisation, especially for high-risk patients, and add to the debate on the best surgical techniques.

## Introduction

Abdominal wall hernias, including groin defects, are a common surgical encounter in the outpatient clinic; however, there has been an increase in emergency presentations in recent years, leading to emergency operations with higher proportions of post-op complications and re-interventions [1]. In contrast, a recent study suggests that postponing elective hernia surgery during the COVID-19 pandemic did not significantly impact emergency presentations or worsen outcomes [2]. While these findings provide valuable insights, they also raise critical questions about the applicability of such conclusions across broader populations, healthcare systems, and non-pandemic conditions. This is particularly important because delayed intervention in hernia cases can still lead to complications that necessitate high-risk emergency surgery with potentially poor outcomes, as shown in this study.

It is generally acknowledged that elective procedures, which enable thorough pre-op optimisation, typically result in the best outcomes for patients with surgical pathologies. Pre-op optimisation, planning, and comprehensive patient assessment are all made possible by elective procedures, and these are essential for reducing surgical risk and maximising recovery and long-term advantages [3].

The aim of this study is to assess the outcomes of patients who presented with abdominal wall hernias on an emergency basis and subsequently underwent surgical repair. This retrospective review spans 10 years and focuses on a variety of abdominal wall hernia types, examining post-op results and the factors associated with increased complications in emergency cases.

Because of the risk of infection and other challenges, mesh is generally avoided in the UK when performing emergency hernia repairs involving the bowel. To lower the chance of recurrence, the European Hernia Society (EHS) guidelines recommend prosthetic reinforcement for ventral hernias larger than 1 cm and for all groin hernias [4]. Additionally, this study aims to assess the results regarding mesh usage patterns and compliance with EHS regulations in emergencies.

## Materials and methods

This is a retrospective study that analyses data retrieved from the medical records of 239 patients, who presented in the Emergency Department of a single District General Hospital with symptomatic abdominal wall hernias requiring urgent surgical intervention in the last decade, from January 2014 to December 2023.

The major cohort characteristics include signs and symptoms, the average age of the patients during the emergency presentation, the type of hernias based on anatomical location, the surgical approach (such as open vs. laparoscopic), and the method of repair (mesh placement vs. primary repair). Furthermore, the length of hospital stay, measured in days from admission to discharge, and post-op complications, including types and severity, have also been included. The study also analyses the outcomes specific to emergency presentations requiring additional surgical interventions, providing information on the factors affecting the prognosis of patients.

The patients were divided into two major groups depending on the types of repairs (primary vs. mesh repair). The two groups were compared based on the type of hernia, recurrence rate, post-op complications, and length of hospital stay. The database was developed and analysed using IBM SPSS Statistics for Windows, Version 26 (Released 2019; IBM Corp., Armonk, NY, USA). Quantitative variables, such as length of hospital stay (days), were summarised by their mean ± SD values. The difference in mean values between the two groups was tested by a two-sample t-test. Qualitative variables, such as type of repair and complications, were presented by their frequencies along with percentages. The association of qualitative variables was tested with the Chi-square test of independence. The results were considered significant at p<0.005.

The inclusion criteria for this study were all male and female patients above 18 years old who presented in the Emergency Department with a symptomatic abdominal wall hernia, including groin hernias, requiring emergency surgery, and patients with comprehensive medical records, including all the cohort characteristics of the study.

The exclusion criteria of the study were all patients below 18 years old, elective hernia repairs, patients managed conservatively and listed for elective hernia repair later, and patients with insufficient post-op follow-up data to assess recurrence or complications.

## Results

**Figure 1:**
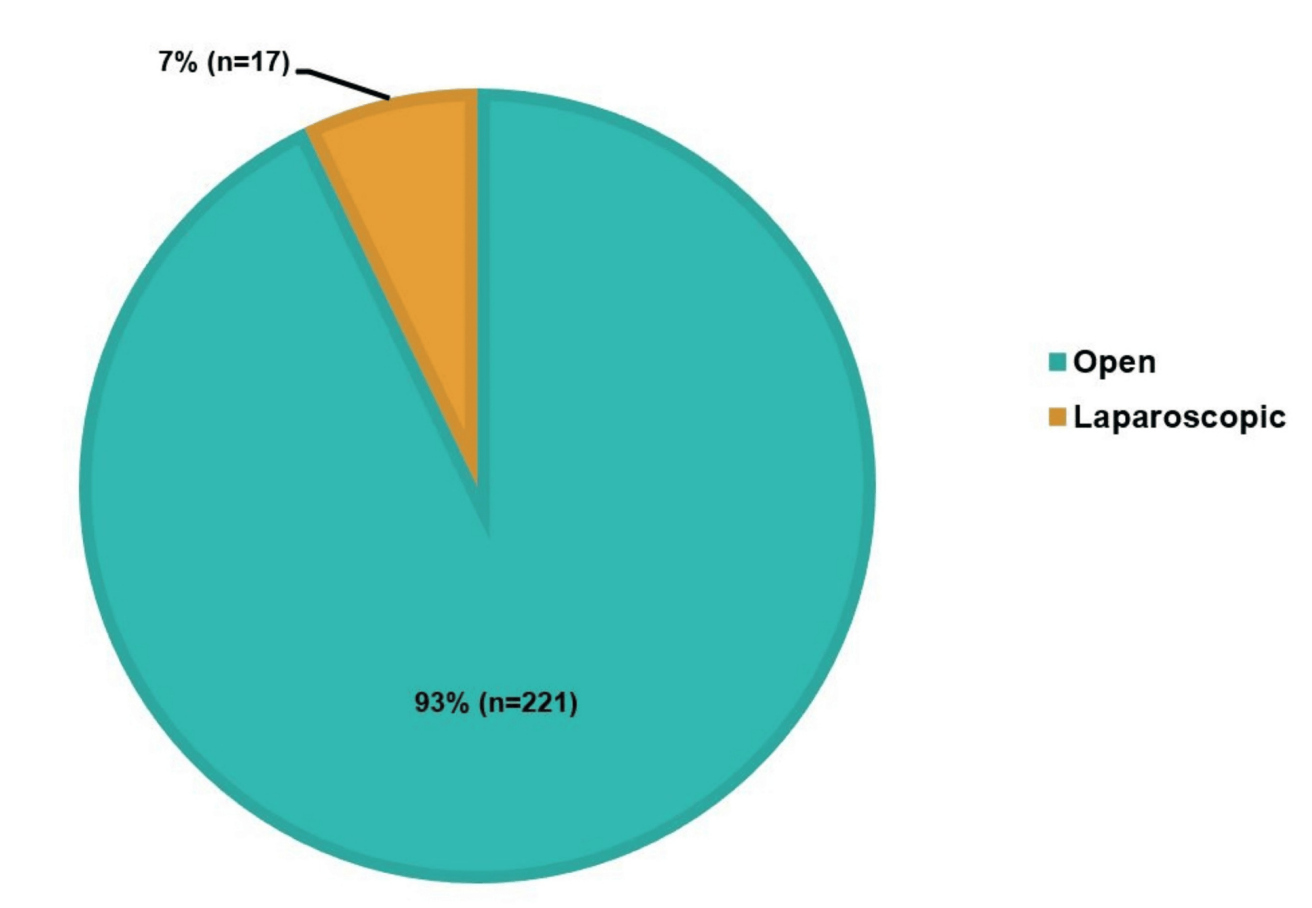
Distribution of open vs. laparoscopic hernia repairs

In the last 10 years, the total number of emergency hernia presentations was 239. The patients who underwent emergency hernia repairs numbered 238, and one patient (n=1) died before surgery. A total of 93% (n=221) of patients underwent the open approach, and 7% (n=17) were treated laparoscopically, as seen in Figure 1.

**Figure 2:**
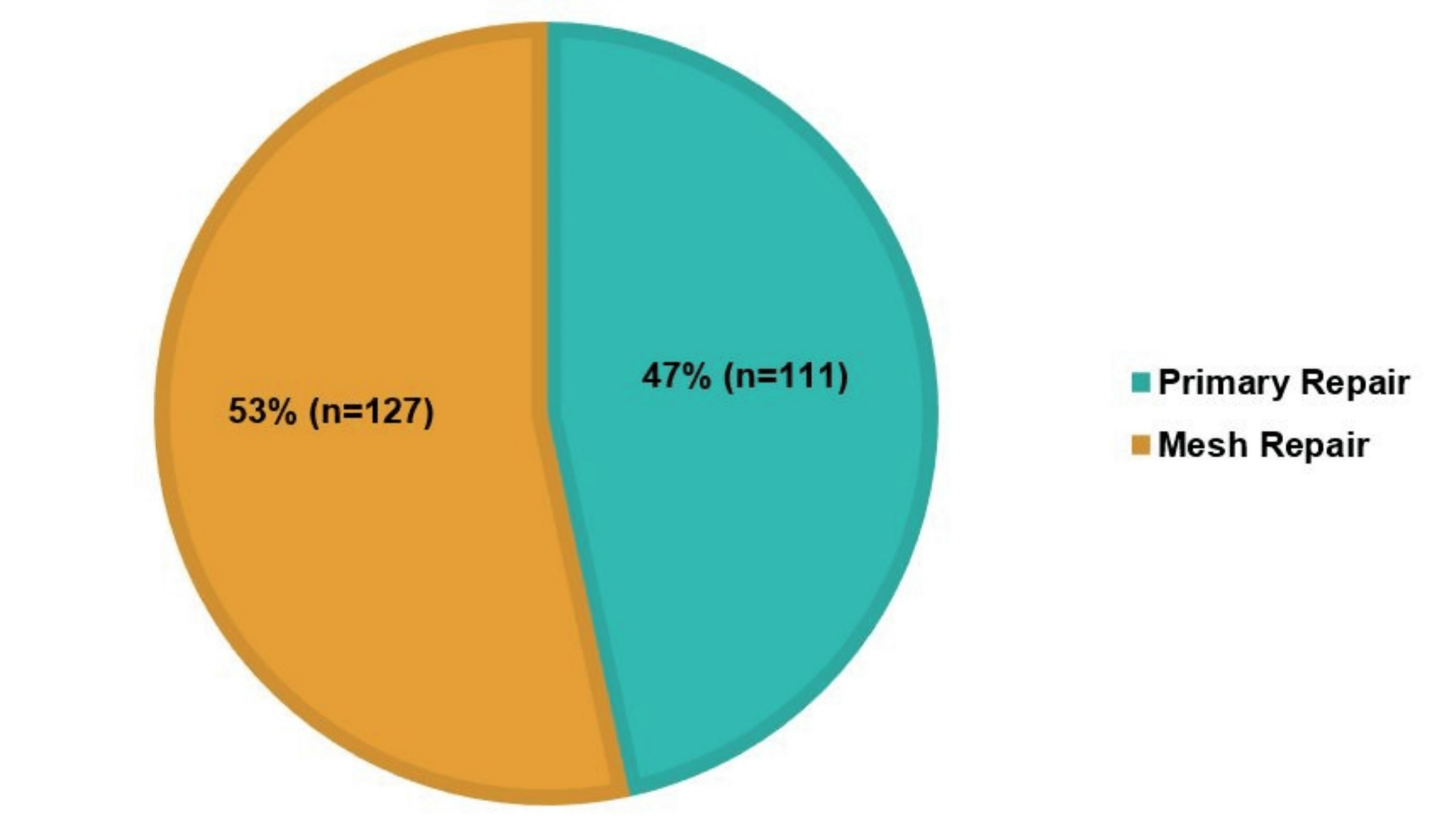
Number of primary repair vs. mesh repair

Primary repair was carried out in 47% (n=111) of cases, while 53% (n=127) of cases involved mesh reinforcement, as shown in Figure 2. The total recurrence rate of hernias was 2% (n=4), with all four occurring in primary repairs. Additionally, in our study, patients who underwent mesh repair had more post-op complications (56%, n=37) compared to primary repair (44%, n=29). Patients who had mesh repair spent an average of eight days in the hospital, whereas those who had primary repair spent nine days, as shown in Table 1, along with the p-value.

**Table 1:** Mean length of stay and p-value between patients that underwent primary vs. mesh repair

Variable	Type of repair	n	Mean±SD	p-value
Length of stay (days)	Mesh	127	8.01±10.21	0.49
	Primary repair	111	9.14±15.13	

In total, 13 different hernia types were observed; however, the most common were inguinal (n=77), umbilical (n=69), and femoral (n=36), respectively. These accounted for over 76% of cases. In terms of primary vs. mesh repair, most of the inguinal hernia repairs were mesh (90%, n=69), and the remaining patients (9%, n=7) underwent primary repair, with one patient dying prior to surgery. While umbilical hernia was the second most common, in contrast to inguinal hernias, most of the umbilical hernia repairs were primary repairs (77%, n=53), and the remaining (23%, n=16) had mesh repairs. For femoral hernias, the third most common, there were an equal number of primary (50%, n=18) and mesh repairs (50%, n=18). The other hernias observed in the study are mentioned in Table 2.

**Table 2:** The type and number of different hernias encountered within the study

Type of hernia	Count	Percentage
Inguinal	77	32.2%
Umbilical	69	28.9%
Femoral	36	15.1%
Incisional	17	7.1%
Epigastric	18	7.5%
Parastomal	9	3.8%
Recurrent	4	1.7%
Spigelian	3	1.3%
Obturator	2	0.8%
Hiatus	1	0.4%
Diaphragmatic	1	0.4%
Internal	1	0.4%
Infected mesh from previous hernia repair	1	0.4%
Total	239	100%

**Figure 3:**
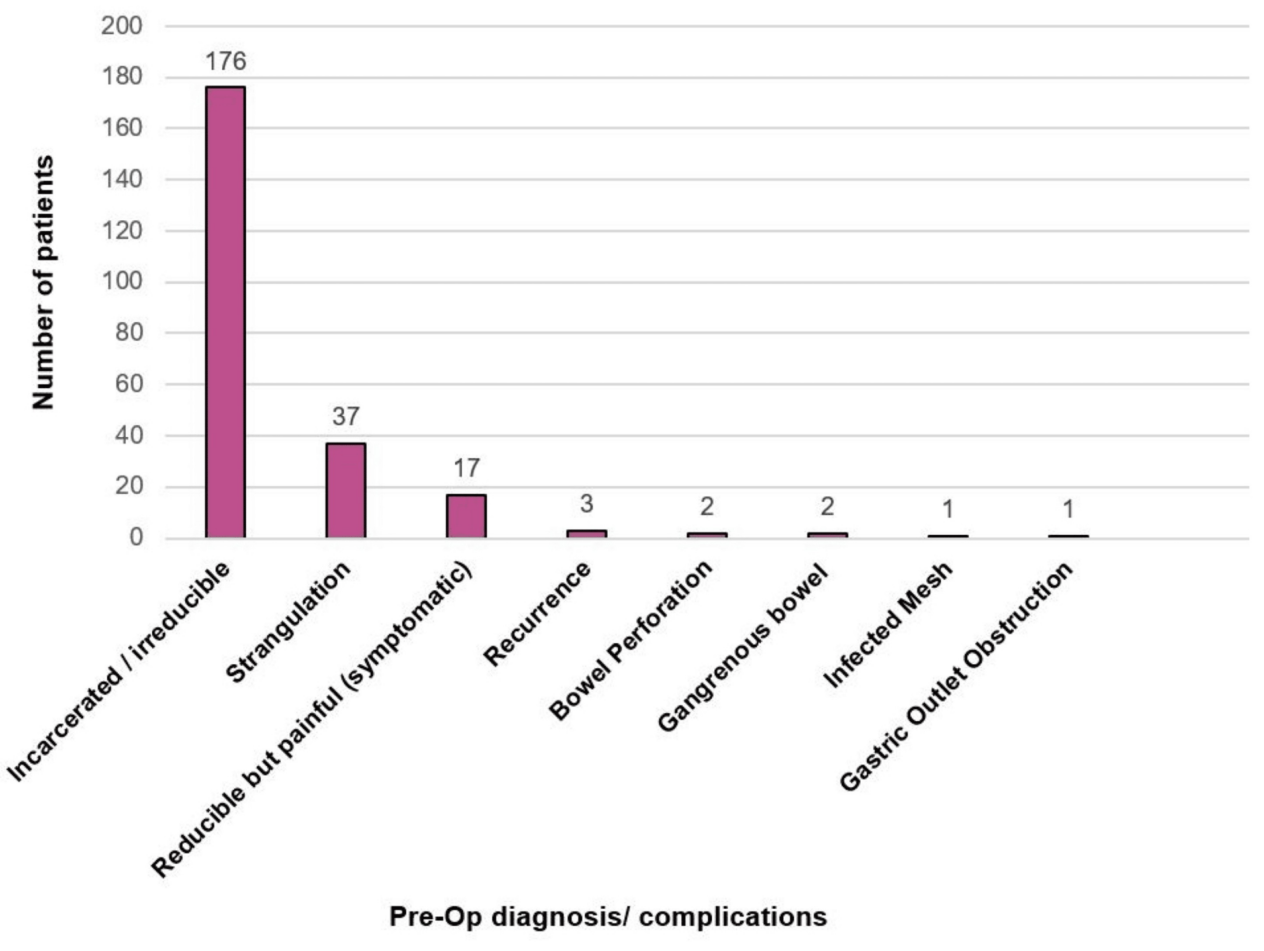
Presenting complaints of patients

The most common presenting complaint was incarcerated or irreducible hernias, accounting for 74% (n=176) of cases. This was followed by strangulation (15%, n=37) and then reducible but painful hernias (7%, n=17). Less frequent pre-op complications included recurrence of a previous hernia (n=3), bowel perforation (n=2), and gangrenous bowel (n=2). There was also one case of infected mesh and one case of gastric outlet obstruction, as shown in Figure 3.

**Figure 4:**
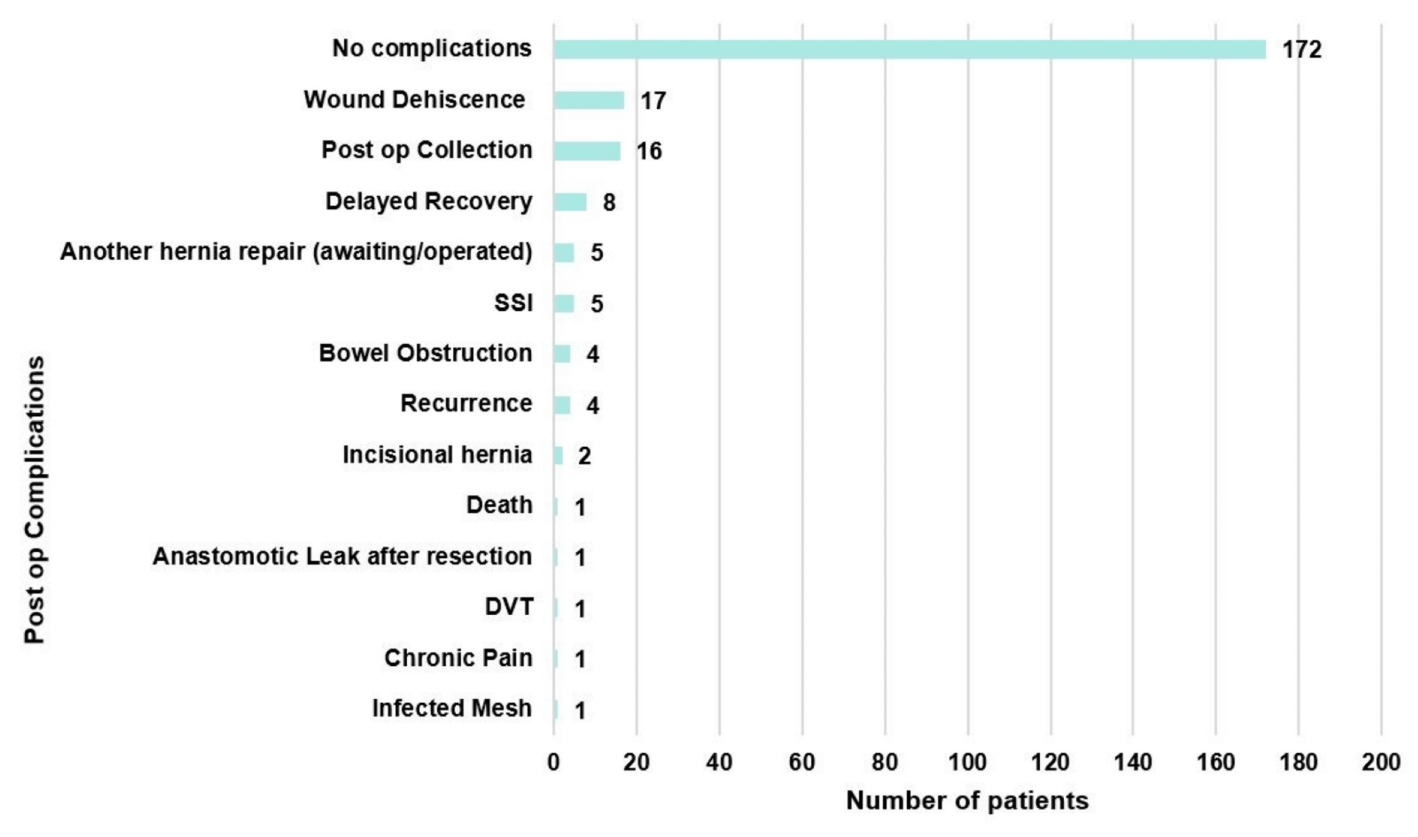
Number and type of post-op complications after emergency hernia surgery SSI, surgical site infection; DVT, deep vein thrombosis

The study reported several post-op complications related to emergency hernia repairs: wound dehiscence, the most common (7%, n=17), followed by post-op collections (7%, n=16), delayed recovery (3%, n=8), another hernia repair (2%, n=5), surgical site infections (SSI) (2%, n=5), and bowel obstruction (1.6%, n=4). Additionally, there were four (n=4) instances of recurrence, two (n=2) patients who developed incisional hernias, one (n=1) case of infected mesh, and one (n=1) case of DVT. Chronic pain was noted in one patient (n=1), and there was one (n=1) anastomotic leak after resection. Unfortunately, death was also reported in one case (n=1). The majority of the cases (n=172, 72%), however, reported no complications. The data for this can be seen in Figure 4. The relation between the type of hernia and post-op complications has been highlighted in the discussion under the heading "Hernia types and post-op complications."

**Figure 5:**
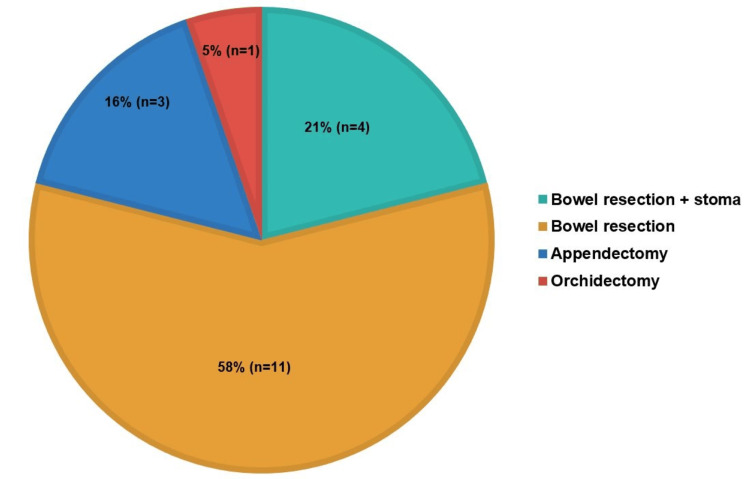
Number of additional surgical interventions

Patients undergoing emergency hernia repairs often face challenging clinical conditions that necessitate additional surgical interventions. A total of 19 patients required further surgical interventions, such as bowel resection (58%, n=11), bowel resection with stoma formation (21%, n=4), appendectomy (16%, n=3), and orchidectomy (5%, n=1), compared to elective repairs. This can be seen in Figure 5. In cases of bowel resection (n=11), femoral hernias (n=5) were associated with pre-op complications as follows: gangrenous bowel (n=1), incarceration (n=2), and strangulation (n=2). Inguinal hernias (n=2) had complications of bowel perforation (n=1) and strangulation (n=1). Incisional hernias (n=2) were linked to gangrenous bowel (n=1) and incarceration (n=1). Epigastric hernia (n=1) and umbilical hernia (n=1) both presented with strangulation as a complication.

For bowel resection with stoma (n=4), inguinal hernias (n=2) and umbilical hernia (n=1) were both complicated by strangulation (n=3), while parastomal hernias (n=1) were associated with bowel perforation. These findings highlight the need for timely elective hernia management to reduce the risk of invasive procedures, extended hospitalisation, and related complications.

## Discussion

In comparison to elective procedures, emergency hernia presentations are linked with much higher rates of morbidity and mortality, as documented in the surgical literature [5]. Emergency hernia repairs frequently involve contamination, incarceration, or strangulation, in contrast to elective repairs, which benefit from pre-op optimisation and controlled conditions. According to the literature and our cohort analysis, these factors increase the risk of post-op complications and longer recovery durations by necessitating rapid surgical intervention, frequently under less-than-ideal circumstances [6]. This study sought to evaluate the outcomes of patients who had undergone emergency surgery over the last decade due to complex, symptomatic abdominal wall hernias.

Surgical techniques (open vs. laparoscopic approach)

The patient outcomes are significantly impacted by the decision between open and laparoscopic hernia repair procedures. The literature indicates that the open technique predominates in emergencies because it is feasible in complex situations, in contrast to elective repair, where laparoscopic repairs are frequently preferred due to their correlation with decreased post-op pain and quicker recovery [7]. Because complex hernias, especially those involving bowel-related complications like strangulation, perforation, and ischaemia, present technical hurdles, most cases in this study (93%, n=221) underwent open surgical repair, rather than laparoscopic repair (7%, n=17). Of the 17 laparoscopic surgeries, six were femoral hernias, four were umbilical hernias, and two were inguinal hernias. The remaining cases were one each of parastomal, epigastric, incisional, ventral, and hiatus hernias. This is consistent with existing literature, which shows that open surgery offers superior access and control when there is tissue ischaemia or bowel involvement, both of which are frequent in emergencies [4]. Furthermore, research has revealed that although laparoscopic repairs may be technically possible in some emergencies, they are often less practical than open surgery due to the increased risk of contamination or inadequate prior patient optimisation [7,8].

Mesh vs. primary repair

The decision between primary repair and mesh reinforcement in emergency hernia cases remains a critical point of debate. In elective hernia repairs, mesh reinforcement is widely favoured due to its role in reducing recurrence rates [9]. In this study, primary repair was carried out in 47% (n=111) of cases, whereas 53% (n=127) of cases involved mesh reinforcement. The type of repair carried out compared to the type of hernia is shown in Table 3. A total of four patients had recurrence, all of whom underwent primary repair, which suggests that utilising mesh repair in emergencies might reduce the rates of recurrence. Mesh reinforcement is advised by the EHS guidelines for elective hernias larger than 1 cm to lower the chance of recurrence; however, it is difficult to follow the guidelines in emergency situations. To weigh the advantages of preventing recurrence against the infection risks associated with mesh use, recent research is evaluating the selective use of mesh in controlled contamination situations, such as partial intestinal obstruction without active ischaemia. Clinical trials are being conducted to establish standards for the safe use of mesh in emergency hernia repairs, making this trend an important area of research [10,11].

**Table 3:** Type of repair compared to the type of hernia

Type of hernia	Type of repair		Total	p-value
	Mesh	Primary repair		
Inguinal	69	7	76	0.001
	90.8%	9.2%	100.0%	
Umbilical	16	53	69	0.001
	23.2%	76.8%	100.0%	
Femoral	18	18	36	1.000
	50.0%	50.0%	100.0%	
Epigastric	10	8	18	0.640
	55.6%	44.4%	100.0%	
Incisional	7	10	17	0.190
	41.2%	58.8%	100.0%	
Para-stomal	3	6	9	0.370
	33.3%	66.7%	100.0%	
Recurrent (inguinal+ventral)	3	1	4	0.430
	75.0%	25.0%	100.0%	
Spigelian	0	3	3	--
	0.0%	100.0%	100.0%	
Obturator	0	2	2	--
	0.0%	100.0%	100.0%	
Internal	0	1	1	--
	0.0%	100.0%	100.0%	
Diaphragmatic	0	1	1	--
	0.0%	100.0%	100.0%	
Removal of mesh+washout	0	1	1	--
	0.0%	100.0%	100.0%	
Hiatus	1	0	1	--
	100.0%	0.0%	100.0%	
Total	127	111	238	--
	53.4%	46.6%	100.0%	--

In this study, bowel resection was performed in 15 (n=15) cases, of which four (n=4) required stoma formation. Among these, perforation was noted in two (n=2) cases, gangrenous bowel in two (n=2), incarceration in three (n=3), and strangulation, the most common pre-op complication, in eight (n=8) cases. Mesh repair was utilised in five (n=5) cases, including four (n=4) for strangulation and one (n=1) for incarcerated hernia; of these, two (n=2) were inguinal hernias, two (n=2) were umbilical hernias, and one (n=1) was a femoral hernia. Primary repair was performed in 10 (n=10) cases, comprising hernias with strangulation (n=4), and two each of incarceration (n=2), gangrenous bowel (n=2), and perforation (n=2).

However, in emergency settings, the choice becomes more complex, particularly when contamination or bowel perforation is present. Infection risks associated with mesh placement in contaminated fields often necessitate primary repair in emergencies, even though this approach is linked with higher recurrence rates over the long term [12]. Still, some studies highlight emerging strategies, such as using biological or absorbable meshes, which show promise in managing emergency repairs with reduced recurrence risks while also managing contamination. This area, however, warrants further investigation to develop protocols that may improve long-term outcomes in emergency hernia repair while minimising complications [13,14].

This study showed that post-op complications were more common in patients undergoing mesh repair compared to primary repair. In the mesh repair group, a total of 37 (56%, n=37) patients had post-op complications, particularly post-op collection (n=12), SSI (n=3), and mesh infection (n=1). In the primary repair group, a total of 29 (44%, n=29) patients had post-op complications, particularly wound dehiscence (n=10) and recurrence (n=4). A meta-analysis published in 2023 compared mesh and non-mesh repairs in patients undergoing emergency groin hernia repair. Their findings coincide with our study, which found that mesh repair was associated with a higher risk of SSIs compared to primary suture repair [15]. The relation between post-op complications and the type of repair can be seen in Table 4.

**Table 4:** Post-op complications compared to the type of hernia repair SSI, surgical site infection; DVT, deep vein thrombosis

Post-op complications	Type of repair		Total	p-value
	Mesh	Primary repair		
No follow-up complication	90	82	172	0.55
	52.3%	47.7%	100.0%	
Wound dehiscence	7	10	17	0.48
	41.2%	58.8%	100.0%	
Post-op collection	12	4	16	0.08
	75.0%	25.0%	100.0%	
Delayed recovery	5	3	8	0.52
	62.5%	37.5%	100.0%	
SSI	3	2	5	0.69
	60.0%	40.0%	100.0%	
Another hernia repair (awaiting/operated)	3	2	5	0.69
	60.0%	40.0%	100.0%	
Recurrence	0	4	4	--
	0.0%	100.0%	100.0%	
Bowel obstruction	2	2	4	--
	50.0%	50.0%	100.0%	
Anastomotic leak after resection	0	1	1	--
	0.0%	100.0%	100.0%	
Chronic pain	1	0	1	--
	100.0%	0.0%	100.0%	
Incisional hernia	1	1	2	--
	50.0%	50.0%	100.0%	
DVT	1	0	1	--
	100.0%	0.0%	100.0%	
Infected mesh	1	0	1	--
	100.0%	0.0%	100.0%	
Death	1	0	1	--
	100.0%	0.0%	100.0%	
Total	127	111	238	--
	53.4%	46.6%	100.0%	--

Hernia types and post-op complications

Specific hernia types, including inguinal, femoral, and ventral hernias, are frequently noted in the literature as high-risk for emergency presentation complications [16]. The most common hernia types observed in this study were inguinal, umbilical, and femoral hernias, with incarcerated or irreducible hernias (n=176) being the most frequent presentation. These conditions are known to increase the risk of complications such as strangulation, perforation, and ischaemia of the bowel, as evidenced by the 37 (n=37) cases of strangulation, two (n=2) cases of bowel perforation, and two (n=2) cases of gangrenous bowel in this cohort. The presence of these conditions often necessitates emergency surgery, which carries a greater risk of post-op complications such as SSIs, wound dehiscence, post-op collection, and recurrence, as compared to those undergoing elective repairs, due to the lack of pre-op optimisation, as both this study and existing literature suggest [17].

In this study, the most common complications following emergency hernia repairs included wound dehiscence (n=17), observed in various hernia types, with femoral and umbilical hernias each accounting for four (n=4) cases. Incarceration was common, occurring in femoral hernias (n=2, with two additional cases involving strangulation), umbilical hernias (n=3, with one case involving strangulation), parastomal hernias (n=2, with one case of perforation), incisional hernias (n=2, with one involving infected mesh), inguinal hernias (n=1, with another case involving recurrence), and epigastric hernias (n=1).

Post-op collections were the second most common complication, with incarceration being the most frequent pre-op issue. Inguinal hernias (n=5) had two cases of incarceration, one case of strangulation, one reducible but painful hernia, and one case of perforation. Umbilical hernias (n=4) involved three cases of incarceration and one case of recurrence. Femoral hernias (n=2), incisional hernias (n=2), epigastric hernias (n=2), and parastomal hernias (n=1) were associated with incarceration.

Delayed recovery was the third most common complication, with incarceration being a frequent pre-op issue. Inguinal hernias (n=4) were associated with three cases of incarceration and one case of strangulation. Umbilical hernias (n=2) and femoral hernias (n=2) each had two and one case of incarceration, respectively, while epigastric hernias (n=1) had one case of strangulation. These findings are consistent with the literature, where emergency surgeries are linked to increased morbidity and mortality due to the urgency, contamination, and often compromised tissue involved [18]. A total of 67 patients had post-op complications. The post-op complications, compared with the type of hernia, can be seen in Table 5.

**Table 5:** Post-op complications compared to the type of hernia SSI, surgical site infection; DVT, deep vein thrombosis

Post-op complications	Type of hernia							
	Epigastric	Femoral	Incisional	Inguinal	Umbilical	Para-stomal	Others	Total
Wound dehiscence	1	4	3	2	4	3	0	17
Post-op collection	2	2	2	5	4	1	0	16
Delayed recovery	1	1	0	4	2	0	0	8
SSI	0	2	0	1	2	0	0	5
Recurrence	1	0	0	1	2	0	0	4
Bowel obstruction	0	1	1	1	0	1	0	4
Another hernia repair (awaiting/operated)	0	2	0	3	0	0	0	5
Anastomotic leak after resection	0	0	0	1	0	0	0	1
Incisional hernia	0	0	0	0	0	1	1	2
DVT	0	0	0	1	0	0	0	1
Infected mesh	0	0	1	0	0	0	0	1
Chronic pain	0	0	0	1	0	0	0	1
Death pre-op	0	0	0	1	0	0	0	1
Death post-op	0	0	0	1	0	0	0	1
Total								67

Mortality and morbidity trends in emergency repairs

Emergency hernia repairs are associated with increased morbidity and mortality, particularly in patients over 70 years of age and those with the American Society of Anesthesiologists (ASA) class III/IV [18]. Older patients, especially those with comorbid conditions such as cardiovascular disease, high BMI, or diabetes, are at heightened risk for post-op complications and mortality following emergency repairs. Literature suggests that, in emergency settings, the physiological stress of advanced disease and urgent surgery, coupled with limited pre-op optimisation, contributes significantly to these unfavourable outcomes [11-14]. This coincides with our study, where the average age of patients undergoing emergency hernia repair was 69, and most of the patients with post-op complications were ASA II/III, as shown in Table 6.

**Table 6:** Comparison of post-op complications with ASA grades Note that the ASA grades were not mentioned or available in the data for 6 patients SSI, surgical site infection; DVT, deep vein thrombosis; ASA, American Society of Anesthesiologists

Post-op complications	ASA grading				Total
	ASA I	ASA II	ASA III	ASA IV	
No follow-up complication	17	69	72	12	170
	10.0%	40.6%	42.4%	7.1%	100.0%
Wound dehiscence	0	8	6	2	16
	0.0%	50.0%	37.5%	12.5%	100.0%
Post-op collection	0	8	7	1	16
	0.0%	50.0%	43.8%	6.3%	100.0%
Delayed recovery	0	0	7	1	8
	0.0%	0.0%	87.5%	12.5%	100.0%
SSI	0	3	1	1	5
	0.0%	60.0%	20.0%	20.0%	100.0%
Another hernia repair (awaiting/operated)	0	3	1	1	5
	0.0%	60.0%	20.0%	20.0%	100.0%
Recurrence	2	0	1	1	4
	50.0%	0.0%	25.0%	25.0%	100.0%
Bowel obstruction	0	2	2	0	4
	0.0%	50.0%	50.0%	0.0%	100.0%
Anastomotic leak after resection	0	1	0	0	1
	0.0%	100.0%	0.0%	0.0%	100.0%
Chronic pain	0	1	0	0	1
	0.0%	100.0%	0.0%	0.0%	100.0%
Incisional hernia	1	0	0	0	1
	100.0%	0.0%	0.0%	0.0%	100.0%
DVT	0	0	0	1	1
	0.0%	0.0%	0.0%	100.0%	100.0%
Death	0	0	1	0	1
	0.0%	0.0%	100.0%	0.0%	100.0%
Total	20	95	98	20	233
	8.6%	40.8%	42.1%	8.6%	100.0%

Delayed presentations often result in severe complications like bowel ischaemia and strangulation, leading to complex surgeries such as bowel resections; therefore, timely intervention is crucial to avoid these complications. As reflected in our study, 15 patients underwent bowel resection, five of whom required a stoma. The fact that two deaths occurred in this study, while unfortunate, emphasises how serious emergency presentations can be, especially for patients who had pre-existing co-morbidities or delayed presentation.

The broader challenge in hernia management is balancing immediate surgical needs with long-term patient outcomes. A study by Fitzgibbons et al. (2006) supports watchful waiting as a safe strategy for men with minimally symptomatic inguinal hernias, allowing for deferral of elective surgery without significant short-term complications [19]; however, the limitation of this study is its short two-year follow-up, which leaves uncertainty about long-term outcomes. This underscores the need for more research to assess the long-term safety of watchful waiting. Our findings emphasise the importance of timely intervention to avoid the risks and additional surgical interventions associated with emergency surgery. Future studies that concentrate on improving surgical results for high-risk hernia patients, streamlining emergency repair procedures, and improving mesh-use approaches could further enhance the standard of hernia care.

Limitations

Since the study is retrospective and limited to a single district general hospital, there is a risk of bias, such as inconsistent data collection and variability in practices and results, which could have been observed in larger healthcare settings. Although the data in the study involved patients’ demographics, it doesn’t provide a detailed analysis of specific co-morbidities, such as cardiovascular disease, diabetes mellitus, obesity, smoking, etc., that could influence surgical outcomes in emergency hernia repairs. While elective cases are excluded, this study does not provide a comparison between emergency and elective repairs or insights into the benefits of timely elective interventions. Furthermore, the study does not address the technical versatility and innovations (e.g., type of mesh, advanced laparoscopic/robotic techniques) in surgical approaches for managing cases.

## Conclusions

In conclusion, emergency hernia repairs are associated with an increased risk of post-op complications, longer length of stay, additional interventions, and, in some high-risk individuals, increased mortality. Emergency interventions take place under challenging conditions, often involving tissue compromise, infection risks, and bowel involvement, which necessitate additional interventions and limit the use of mesh, compared to elective hernia repairs. This study highlights the importance of elective hernia management in preventing emergency presentations from leading to adverse outcomes and reducing healthcare costs by decreasing the need for complex interventions, which are common in emergency cases. To enhance care in urgent hernia repairs, more research should concentrate on improving surgical techniques and emergency protocols.
